# Domain-specific cognitive course in schizophrenia: Group- and individual-level changes over 10 years

**DOI:** 10.1016/j.scog.2022.100263

**Published:** 2022-06-22

**Authors:** Camilla Bärthel Flaaten, Ingrid Melle, Thomas Bjella, Magnus Johan Engen, Gina Åsbø, Kristin Fjelnseth Wold, Line Widing, Erlend Gardsjord, Linn-Sofie Sæther, Merete Glenne Øie, Siv Hege Lyngstad, Beathe Haatveit, Carmen Simonsen, Torill Ueland

**Affiliations:** a NORMENT, Division of Mental Health and Addiction, Oslo University Hospital & Institute of Clinical Medicine, University of Oslo, P. O. box 4956 Nydalen, 0424 Oslo, Norway; b Department of Psychology, University of Oslo, Forskningsveien 3A, 0373 Oslo, Norway; c Division of Mental Health and Addiction, Nydalen DPS, Oslo University Hospital, P.O. Box 4956 Nydalen, 0424 Oslo, Norway; d Division of Mental Health and Addiction, Unit for Early Intervention in Psychosis, Oslo University Hospital, P.O. Box 4956 Nydalen, 0424 Oslo, Norway; e Early Intervention in Psychosis Advisory Unit for South East Norway, Division of Mental Health and Addiction, Oslo University Hospital, P. O. box 4956 Nydalen, 0424 Oslo, Norway

**Keywords:** Cognition, Long-term cognitive development, First-episode schizophrenia

## Abstract

Cognitive impairments in schizophrenia are well-documented, present across several cognitive domains and found to be relatively stable over time. However, there is a high degree of heterogeneity and indications of domain-specific developmental courses. The present study investigated the 10-year cognitive course in participants with first-episode schizophrenia (FES) and healthy controls on eight cognitive domains and a composite score, looking at group- and individual-level changes.

A total of 75 FES participants and 91 healthy controls underwent cognitive assessment at baseline and follow-up. Linear mixed models were used for group-level analyses and reliable change index (RCI) analyses were used to investigate individual change. The prevalence of clinically significant impairment was explored at both time points, using a cut-off of < −1.5 SD, with significant cognitive impairment defined as impairment on ≥2 domains.

Group-level analyses found main effects of group and time, and time by group interactions. Memory, psychomotor processing speed and verbal fluency improved, while learning, mental processing speed and working memory were stable in both groups. FES participants showed deteriorations in attention and cognitive control. Individual-level analyses mainly indicated stability in both FES and controls, except for a higher prevalence of decline in cognitive control in FES. At baseline, 68.8 % of FES participants had clinically significant impairment, compared to 62.3 % at follow-up.

We mainly found long-term stability and modest increases in cognition over time in FES, as well as a high degree of within-group heterogeneity. We also found indications of deterioration in participants with worse cognitive performance at baseline.

## 1 Introduction

Schizophrenia is widely considered a neurodevelopmental disorder (Melle, 2019; Rund, 2018), but there is still some contention as to whether the cognitive course is best described in terms of developmental deficits, lag, or deterioration (Panayiotou et al., 2020; Zanelli et al., 2019). Deficits are found in several cognitive domains, including processing speed, attention, working memory, verbal learning and memory, and executive functions (Fatouros-Bergman et al., 2014; Keefe and Fenton, 2007), with significant heterogeneity in the level of impairment (Weinberg et al., 2016). There are several longitudinal studies on cognition in schizophrenia, and findings from these are somewhat diverging (Shmukler et al., 2015). Some find long-term improvements (Fu et al., 2018), some find deterioration (Bonner-Jackson et al., 2010), however with most studies showing group-level stability (Øie et al., 2021; Bergh et al., 2016; Hoff et al., 2005; Mesholam-Gately et al., 2009; Townsend and Norman, 2004). Importantly, very few studies have long-term follow-ups, a first-episode sample, broad test-batteries and healthy controls Hoff et al., 2005; Zanelli et al., 2019).

Findings are also inconsistent regarding the trajectories of specific cognitive functions. For example, one study observed developmental lag for processing speed, attention, visuospatial ability, and working memory, as opposed to stable deficits in knowledge acquisition and reasoning (Reichenberg et al., 2010). Zanelli et al. (2019), on the other hand, found stability in processing speed and executive functions, and declines in verbal reasoning and memory. Accordingly, studies should be cognizant of developmental differences between specific cognitive functions and examine domain-level trajectories (Mesholam-Gately et al., 2009; Shmukler et al., 2015).

While there is a large degree of heterogeneity in cognitive trajectories (Heilbronner et al., 2016; Seidman and Mirsky, 2017), most studies are based on group-level analyses. Individual differences in course can, however, be detected using a reliable change index (RCI) to identify participants with changes beyond those expected from practice effects (Gray et al., 2014). This approach has been used to study group- and individual-level effects in development of executive functioning in first-episode psychosis (Haatveit et al., 2015). Alternatively, as frequently done in studies of mild cognitive impairment, the prevalence of cognitive impairment can be explored using set criteria, typically 1–2 SD below norm mean (Kochan et al., 2010). For instance, using a threshold of −1 SD, approximately 80 % of participants with schizophrenia can be defined as cognitively impaired (Keefe and Fenton, 2007). It is recommended that several domains are included in impairment criteria, as this affords a broader scope and more reliable estimations than single-measure approaches (Keefe and Fenton, 2007; Kochan et al., 2010).

The aim of the present study was to investigate the cognitive course in FES over a 10-year period, compared to a healthy control group. First, we investigated group-level differences in a range of cognitive domains and a cognitive composite score from baseline to follow-up. Based on the existing literature, we expected stable deficits relative to controls in the cognitive composite, with an open hypothesis concerning possible domain-specific changes. Further, we explored individual level differences, in terms of prevalence of both clinically significant cognitive impairment and clinically significant change.

## 2 Methods

### 2.1 Participants

Participants were recruited at the Norwegian Center for Mental Disorder Research (NORMENT) and are part of the Thematically Organized Psychosis (TOP)-sample. Participants with first-episode schizophrenia spectrum disorders were recruited from hospitals and outpatient clinics in the larger Oslo area, and Innlandet Sykehus HF. Baseline assessments started in 2005 and ran consecutively to 2012. Follow-up assessments ran consecutively from 2015 to 2021. Baseline assessments were done when participants were clinically stable and within 12-months of first treatment for psychosis. Control participants were randomly selected from the same catchment area based on statistical records.

**Fig. 1:**
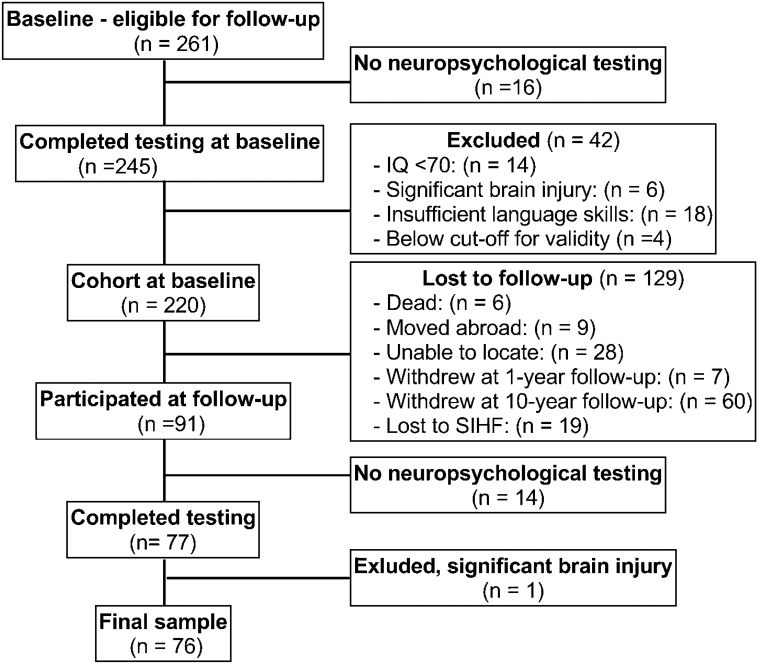
Participant flow. Participant flow and causes of attrition. SIHF: Sykehuset Innlandet HF (Innlandet hospital).

Participants completing baseline assessments and eligible for participation in the follow-up study comprised 261 participants diagnosed with DSM-IV schizophrenia-spectrum disorders at baseline, (70.5 % schizophrenia, 16.7 % schizoaffective disorder, and 12.8 % schizophreniform disorder), and 164 healthy controls. Participant flow is shown in Fig. 1. The retention rate for the FES sample was 35.5 %. Exclusion criteria for both groups were clinically significant head injury, IQ < 70 and insufficient Norwegian-language proficiency for cognitive testing. Controls were screened for history of drug abuse the last 12 months, severe mental illness, and first-degree relatives with severe mental illness. Fifty-six controls were lost to follow-up, rendering the retention rate 65.9 %. Three were excluded due to an acquired brain injury and 10 excluded due to psychiatric illness with onset during follow-up. Our follow-up sample consisted of 76 FES participants and 108 controls. Participants signed written consent, and the study was approved by the Regional Committee for Medical Research Ethics and the Norwegian Data Inspectorate.

### 2.2 Clinical measures

The DSM-IV criteria were used for determining diagnoses, based on the Structured Clinical Interview for DSM-IV axis 1 (SCID-I; First et al., 1995) with additional information from medical records. Trained clinical psychologists and medical doctors performed the assessments under supervision of senior scientists and clinicians. The Global Assessment of Functioning, split version, Symptom scale (GAF-S) was used at both time-points to measure global symptom severity, and the GAF-F was used to measure global functioning (Pedersen et al., 2007). Psychotic symptoms were assessed using the Positive and Negative Syndrome Scale (PANSS; Kay et al., 1987) and scores are reported using the Wallwork five-factor model (Wallwork et al., 2012).

### 2.3 Cognitive measures

Psychologists administered neuropsychological assessments of FES participants, and trained personnel assessed controls. Test users were calibrated and supervised by a neuropsychologist. The battery included the following measures: California Verbal Learning Test (CVLT-II; Delis et al., 2000), Logical Memory (LM) from the Wechsler Memory Scale (Wechsler, 1987), Color-Word Association Test (COWAT) and Verbal Fluency (VF) from the Delis-Kaplan Executive Functioning System (Delis et al., 2001), and Digit Span, Letter-Number Sequencing and Digit-Symbol Coding from the Wechsler Adult Intelligence Scale (Wechsler, 1997).

At both time-points, IQ was measured with four subtests from the Norwegian version of the Wechsler Abbreviated Scale of Intelligence (WASI; Wechsler, 2011). Premorbid IQ was estimated using the National Adult Reading Test, Norwegian version (NART; Blair and Spreen, 1989). Raw scores were converted to z-scores based on controls at baseline. Using means of subtest scores, we calculated eight theory-based domain-scores:

➢ Learning: CVLT-II list A total recall, LM sum trial 1
➢ Memory: CVLT-II long-delay free recall, LM sum trial 2
➢ Attention: Digit span
➢ Psychomotor processing speed: Digit symbol coding
➢ Mental processing speed: COWAT Color naming, Reading
➢ Working memory: Letter-number sequencing
➢ Verbal fluency: VF FAS, Categories, Switching
➢ Cognitive control: COWAT Interference, Switching

A cognitive composite was calculated using the mean of all domain scores.

### 2.4 Statistical analyses

This work used the TSD (Tjeneste for Sensitive Data) facilities, owned by the University of Oslo, operated, and developed by the TSD service group at the University of Oslo, IT-Department (USIT). Statistical analyses were performed using the Statistical Package for the Social Sciences (SPSS) for Windows, version 28. Raincloudplots were created using RStudio version 1.3.1093 (RStudio Team, 2020) with the ggplot2, dplyr, and Raincloudplots packages (Wickham et al., 2016; Wickham et al., 2018; Allen et al., 2021). One outlier was removed from the FES group, resulting in a final sample of 75. Linear mixed models were used for analyses of changes in each domain, first with group comparisons and next with separate growth curves for each group. Intercept was entered as random with covariance structure set to identity, estimates were based on maximum likelihood and we covaried for age. The group model used the following formula:

$$Y_{\mathrm{ij}}=\left(\beta_{1}+b_{\text{1j}}\right)+\beta_{2}*\text{time}+\beta_{3}*\text{group}+\beta_{4}*\mathrm{age}+\beta_{5}*\text{time}*\text{group}+\beta_{6}*\text{time}*\mathrm{age}+e_{\mathrm{ij}}$$

where Y_ij_ is score for person *i* = 1…167 at year j = 0…10, β signifies fixed effects, b signifies random effects, and e is the error term. Growth curves used the same formula excluding the group effect terms.

To explore individual changes, we calculated a regression-based RCI from domain and composite scores, allowing for correction for regression towards the mean and practice effects (Gray et al., 2014). First, regression models predicting change based on baseline scores were run on each group, with group mean change and baseline mean as reference points. Second, parameters from the models were entered in the formula described by Gray et al. (2014) to calculate individual upper and lower limits of the RCI with a 90 % confidence interval.

To assess the prevalence of cognitive impairment, we analyzed scores at baseline and follow-up based on criteria using a combination of severity and extent of impairment (Kochan et al., 2010). We chose a cut-off of −1.5 SD for clinically significant impairment at the domain-level and impairment on at least 2 domains as the threshold for cognitive impairment. Our cut-off was set to ensure stringency regarding the severity of impairment relative to those commonly found in this population (Keefe and Fenton, 2007), thus identifying individuals with both marked and widespread impairments (Kochan et al., 2010).

## 3 Results

Premorbid and current IQ, demographic and clinical characteristics are presented in Table 1. The FES sample was significantly younger than controls and, expectedly, had fewer years of education and lower IQ.

**Table 1:** Demographics and clinical characteristics.

M (SD)	Schizophrenia (*N* = 75)		Healthy controls (*N* = 108)	Group comparisons	
				t/χ^2^	p
Demographics					
Age	26.04 (7.66)		30.92 (7.42)	4.30	p < .000
Gender M (%)	41 (52.6)		61 (57.5)	0.45	p = .502
Education	12.13 (2.18)		14.29 (2.13)	6.68	p < .000
NART premorbid IQ	110.59 (7.73)		113.46 (5.98)	−2.55	p = .012
WASI baseline IQ	105.19 (13.99)		114.90 (8.57)	−5.08	p < .000
WASI follow-up IQ	111.53 (13.18)		117.82 (8.96)	−3.63	p < .000

Clinical characteristics	Baseline	Follow-up			
GAF S	38.55 (10.58)	53.65 (15.82)		−7.56	p < .000
GAF F	39.69 (10.39)	55.00 (16.00)		−7.72	p < .000
PANSS positive	11.76 (4.03)	8.39 (3.95)		5.19	p < .000
PANSS negative	14.75 (6.66)	11.55 (5.91)		3.95	p < .000
PANSS disorganized	6.04 (2.71)	4.84 (2.32)		3.72	p < .000
PANSS excited	6.21 (2.23)	4.84 (1.49)		4.66	p < .000
PANSS depressed	8.76 (3.10)	6.60 (2.71)		5.10	p < .000

WASI: Wechsler Abbreviated Intelligence Scale, NART: National Adult Reading Test, GAF S: Global assessment of Functioning Scale - symptoms, GAF F: Global Assessment of Functioning Scale – functioning, PANSS: Positive and Negative Syndrome Scale, scores are grouped according to the Wallwork five-factor model.

### 3.1 Group-level analyses

**Fig. 2:**
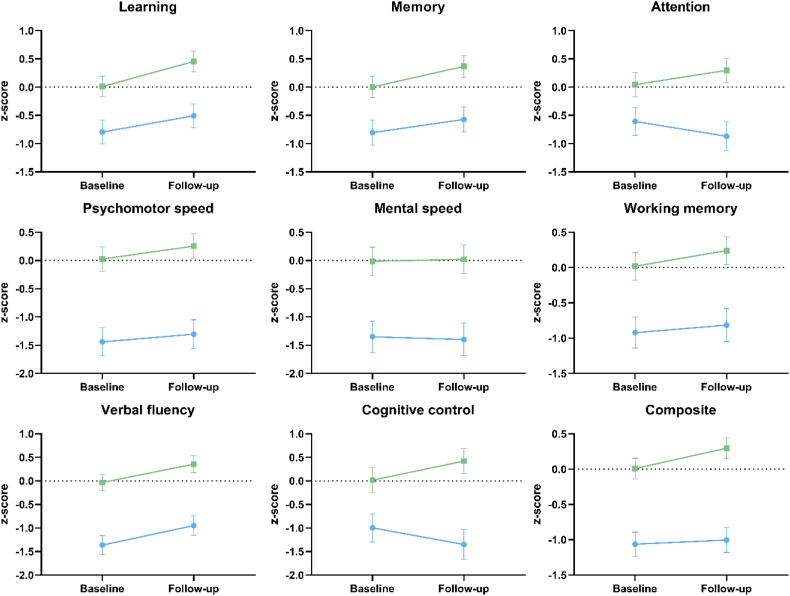
Estimated marginal means of cognitive domain- and composite scores at baseline and follow-up. Line graphs showing group-level changes in each domain from baseline to follow-up. The schizophrenia group is represented by blue lines, and healthy controls are represented by green lines. (For interpretation of the references to color in this figure legend, the reader is referred to the web version of this article.)

Estimated marginal means of domain and composite scores are shown in Fig. 2. We found increases for memory (*t* = −2.07, p = .044), psychomotor speed (*t* = −2.45, p = .013), verbal fluency (*t* = −5.52, p < .001) and the composite (*t* = 3.01, p = .003). Age-effects were found for attention (*t* = −2.28, p = .023), psychomotor speed (t = −2.45, p = .015), cognitive control (*t* = −2.50, p = .013) and the composite (t = −2.07, p = .039), with lower scores for older individuals. We found group*time interactions for attention (*t* = −3.42, p < .001), cognitive control (*t* = −3.50, p < .001) and the cognitive composite (*t* = −2.85, p = .005). While controls improved on all these measures, the FES group had slight deteriorations in attention and cognitive control and a more level slope for the composite. Age*time interactions were found for fluency (*t* = 3.60, p < .001) and for the composite (*t* = 2.59, *p* = .011), showing lower gains with age. Group differences were significant for all domains at p < .001.p

**Fig. 3:**
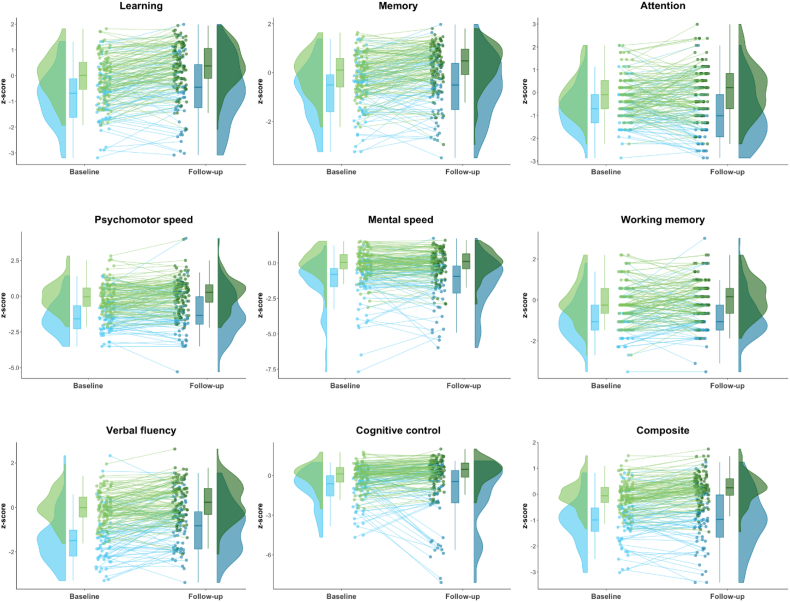
Distribution of domain and composite scores at baseline and follow-up. Distribution of scores from baseline to follow-up are presented in Raincloudplots, showing dispersion, box plots and individual trajectories. The schizophrenia group is shown in blue, and healthy controls are shown in green. (For interpretation of the references to color in this figure legend, the reader is referred to the web version of this article.)

Growth curves of each group showed increases in learning, memory, attention, fluency, control, and composite score for controls, while the FES group only improved on psychomotor speed and fluency. Visualization of the score-distribution and individual trajectories are shown in Fig. 3, illustrating the high heterogeneity in scores and slopes.

### 3.2 Individual-level analyses

RCIs showed a high percentage of stability over the follow-up period. For controls, stability ranged from 87.7 % for attention to 94.1 % for memory, while in the FES group stability ranged from 86.3 % for cognitive control to 95.7 % for psychomotor speed. A similar number of participants in both groups had significant changes, for most domains and the composite (see Table 2). For cognitive control, 13.7 % in the FES group and 8.6 % of the controls declined over time. None of the FES participants improved on this function, as opposed to 1.9 % of the controls.

**Table 2:** Mean change and percentage of participants with clinically significant changes.

	Schizophrenia				Healthy controls			
	Ȳ	B	Decrease	Increase	Ȳ	B	Decrease	Increase
Learning	0.307	−0.063	4.6	3.1	0.444	−0.346	5.9	3.9
Memory	0.271	−0.222	7.7	3.1	0.349	−0.262	3.9	2.0
Attention	−0.249	−0.280	1.4	8.3	0.218	−0.271	5.7	6.6
Psychomotor ps	0.195	−0.111	1.4	2.9	0.199	−0.217	4.8	3.8
Mental ps	−0.035	−0.278	6.8	2.7	0.011	−0.288	2.8	3.8
Working memory	0.118	−0.350	5.8	5.8	0.227	−0.439	2.8	3.8
Fluency	0.471	−0.438	5.7	5.7	0.330	−0.228	5.9	6.0
Cognitive control	−0.298	−0.204	13.7		0.340	−0.286	8.6	1.9
Composite	0.075	−0.001	4.9	3.3	0.264	−0.100	6.1	3.1

Change scores based on the RCI regression analyses, with baseline score as predicted variable and group mean change as dependent. Ȳ = mean change, B = unstandardized regression coefficient, Decrease = percentage with significant decreases, Increase = percentage with significant increases.

**Fig. 4:**
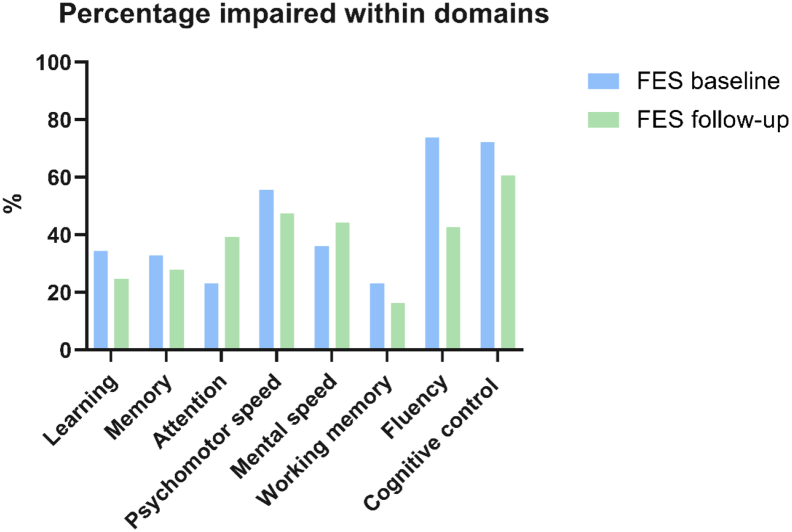
Percentage of participants with impairment on each domain. Percentage of participants reaching cut-off for impairment (−1.5 SD below mean) on each cognitive domain. FES: first episode schizophrenia.

Respectively, 68.8 % and 62.3 % in the schizophrenia group had clinically significant cognitive impairment at baseline and follow-up (see Fig. 4).

Of FES participants meeting criteria for clinically significant impairment at baseline, 57.2 % improved beyond threshold on at least one domain, whereof 16.7 % were no longer impaired. However, 33.3 % of the impaired group at baseline reached the criteria for additional domains, with 4.9 % showing impairment on all domains at follow-up. In the unimpaired group, 31.6 % improved and 26.3 % declined, of which 15.8 % met criteria for clinical impairment at follow-up.

## 4 Discussion

This 10-year follow-up study found significantly lower scores in FES participants compared to controls on all domains at both time points, with stability or modest increases in most domains. We found group-level stability in verbal learning, mental processing speed and working memory, and improvements mirroring those found for controls for memory, psychomotor speed, and fluency. However, interaction effects indicated declines in the FES group relative to controls for attention and cognitive control. Separate growth curves indicated that controls improved in memory and the composite score, both groups made gains in fluency, while the FES group was driving the increase in psychomotor speed.

At the individual level, the percentage of participants with significant changes within domains was low, except for cognitive control, where 13.7 % of participants declined while none improved significantly. Most FES participants had clinically significant impairment at baseline. Improvements were observed in most participants with cognitive impairment, although only 16.7 % improved beyond threshold. A small group of participants with widespread impairments at baseline had clinically significant impairment on all domains at follow-up.

We observed particularly marked and prevalent impairments for speed-dependent measures, in accordance with literature emphasizing deficits in processing speed (Ojeda et al., 2012; Dickinson et al., 2007). Despite impairments at baseline, performance improved, in contradiction to previous findings of deterioration even in cognitively intact individuals (Mesholam-Gately et al., 2009). The impairments in verbal fluency are in line with a hypothesis of a compromised semantic system underlying cognitive impairment in schizophrenia (Bokat and Goldberg, 2003). Nevertheless, the largest improvements were found in fluency. Although a previous study found stability in fluency over a one-year follow-up (Foussias et al., 2021), this is not necessarily in contradiction to findings of long-term improvements. One could speculate that the strong effect in verbal fluency is due to its demands on processing speed and vocabulary, both of which improved in the FES group.

In contrast, FES participants worsened on attention and cognitive control at follow-up, as opposed to findings of improvement in a previous FEP study (Mollon et al., 2020). The reductions in cognitive control were notable, as this was also the domain with the largest percentage of clinically significant decline (13.7 %). As cognitive control contains both processing speed- and executive elements, decrements are somewhat inconsistent with the observed improvements in verbal fluency. However, in addition to cognitive flexibility, cognitive control uniquely contains a strong element of response inhibition, a function consistently found to be impaired in schizophrenia (Thai et al., 2019).

Our results are not in clear support of any single overall hypothesis of developmental deficit, lag, or deterioration. We observed group-level domain-specific trajectories of both improvements, stability, and deterioration, suggesting that long-term cognitive development is driven by several processes (Meier et al., 2014). Group-level analyses of domains mainly suggest stable developmental deficits, with minor fluctuations. There are however some indications of lag when comparing groupwise growth curves, as controls were driving improvements on most domains. Notably, few previous studies support a global neurodegenerative hypothesis, although a rare 20-year follow-up study did find deteriorations across domains (Fett et al., 2020), as opposed to another study showing stability followed by improvements (Bonner-Jackson et al., 2010). Nevertheless, neurodevelopmental and neurodegenerative models need not be mutually exclusive; changes could reflect parallel processes including both atypical development and accelerated cognitive aging (Kobayashi et al., 2014).

Additionally, a large degree of heterogeneity in cognitive trajectories is apparent from the data. Variability in FES scores increased over time, with a notable minority deteriorating, a pattern not found in the controls. As seen by the analyses of clinically significant change and impairment, there appears to be a subgroup with marked and widespread impairments over time. This is consistent with indications of a subgroup with generalized cognitive impairments reported by others (Ammari et al., 2010; Habtewold et al., 2020), and partly consistent with findings of deterioration in more cognitively compromised individuals (Albus et al., 2019). These individuals could be at risk for a poor cognitive outcome and might profit from cognitive remediation as an early intervention (Habtewold et al., 2020).

Our findings are in line with existing literature demonstrating separate trajectories for specific cognitive domains, albeit with differences regarding domain-level effects. This is possibly due to sample characteristics, test-batteries, moderating factors including age and baseline performance, and the effect of non-cognitive factors (McCleery and Nuechterlein, 2019; Moritz et al., 2021; (Heilbronner et al., 2016). The results however clearly demonstrate the importance of investigating domain-level trajectories. A future goal would be to disentangle how subgroup- or individual factors interact with the development of specific cognitive functions.

### 4.1 Strengths and limitations

Strengths of this study include a well-described FES sample followed longitudinally from baseline to 10-year follow-up, as well as the inclusion of a representative sample of healthy controls from the same city, using a comprehensive neuropsychological test battery at both time-points. This enabled investigation of long-term cognitive course in FES compared to the general population, thus providing an updated and appropriate population norm for the domain scores.

An important limitation of this study is the high attrition rate. Although at the level seen in other longitudinal studies using extensive translational protocols (Leeson et al., 2011; Siegel et al., 2006; Velthorst et al., 2017), it resulted in a relatively small follow-up sample, with the risk of underpowered analyses. It also poses a risk for the representativeness of our sample, which could have consequences for conclusions regarding clinical implications. Specifically, completers could be healthier, or we could lose participants at both extremes of outcome, which in turn could affect our estimates of cognitive course. However, we did not observe any differences between participants included and those lost to follow-up in baseline clinical characteristics, IQ, or cognitive measures and visual inspection revealed no marked differences in cognitive variability. There were no significant differences in change-scores between those with high (>0) and low (<−1.5) baseline composite scores. We chose a stringent RCI, which may have conflated practice effects with developmental changes. This method is primarily made for intervention studies and not studies on long-term development. Regardless, this measure has the advantage of accounting for regression to the mean allowing for larger uncertainties at the extremes. Nonetheless, this should be considered when interpreting the results.

## 5 Conclusion

We found stable or improved cognitive trajectories in FES over time, though with some indications of domain-specific effects, as well as substantial heterogeneity. Group-level analyses showed improvements in memory, psychomotor processing speed, verbal fluency, and the cognitive composite. Learning, mental processing speed, and working memory remained stable, while the FES participants deteriorated in attention and cognitive control. The FES group scored lower than controls on all domains. Individual-level analyses mainly showed stability, except for a higher occurrence of decline in cognitive control in FES. Most FES participants reached the criteria for clinically significant cognitive impairment. The results suggest that individuals with severe and widespread impairments in the early illness-phase may be at risk for further decline and may profit from tailored treatment to improve cognitive outcomes.

## Author statement and funding source

All the co-authors of our submitted manuscript “Domain-specific cognitive course in schizophrenia: group- and individual-level changes over 10 years” have seen and approved the final version of the submitted manuscript. This manuscript is an original work that has not been previously published and is not considered for publication in other journals. This research was supported by the Regional Health Authority of Eastern Norway, projects # 2014/1022015/0882018/093 and the Norwegian Research Council, projects #181831213974287714.

## Declaration of competing interest

The authors have no conflicts of interest to declare.

## Appendix A Supplementary data

Supplementary tables Image 1
