# Supplementary material for: Domain-specific cognitive course in schizophrenia: Group- and individual-level changes over 10 years
Source: Schizophr Res Cogn. 2022 Jun 22;30:100263. doi: 10.1016/j.scog.2022.100263 (PMC9240854; doi:10.1016/j.scog.2022.100263)
Supplement: Supplementary file 1 — Supplementary tables [file mmc1.docx]

**Supplementary**

Table 3: Variability in completers vs non-completers

| Domain | Group | SD | Range | Interquartile range |
| --- | --- | --- | --- | --- |
| Learning | Completers | .99 | 4.52 | 1.28 |
|  | Non-completers | .94 | 4.11 | 1.27 |
| Memory | Completers | 1.09 | 4.88 | 1.50 |
|  | Non-completers | 1.06 | 4.54 | 1.49 |
| Attention | Completers | .95 | 4.93 | 1.23 |
|  | Non-completers | .87 | 3.69 | .92 |
| Psychomotor speed | Completers | 1.11 | 4.94 | 1.45 |
|  | Non-completers | 1.25 | 4.94 | 1.93 |
| Mental speed | Completers | 1.71 | 8.89 | 1.65 |
|  | Non-completers | 1.73 | 9.66 | 2.09 |
| Working memory | Completers | .97 | 5.30 | .82 |
|  | Non-completers | .77 | 3.67 | .82 |
| Fluency | Completers | 1.07 | 5.71 | 1.17 |
|  | Non-completers | .96 | 4.92 | 1.34 |
| Cognitive control | Completers | 1.56 | 8.50 | 1.92 |
|  | Non-completers | 1.73 | 8.04 | 2.00 |
| Composite score | Completers | .86 | 3.85 | 1.13 |
|  | Non-completers | .85 | 4.53 | 1.06 |

*Measures of variability in the sample of completers compared to non-completers in the schizophrenia group for each domain and the composite score.*

Table 4: Estimated marginal means from group-level mixed model analyses

|  |  |  |  | | 95% CI | |  |
| --- | --- | --- | --- | --- | --- | --- | --- |
|  | Group | EM | SE | Lower | | Upper | |
| Learning |  |  |  |  | |  | |
| HC | Baseline | .011 | 0.092 | -0.169 | | 0.192 | |
|  | Follow-up | .455 | 0.092 | 0.273 | | 0.637 | |
| FES | Baseline | -.794 | 0.107 | -1.005 | | -0.584 | |
|  | Follow-up | -.508 | 0.108 | -0.720 | | -0.296 | |
| Memory |  |  |  |  | |  | |
| HC | Baseline | .003 | 0.096 | -0.186 | | 0.192 | |
|  | Follow-up | .368 | 0.096 | 0.178 | | 0.558 | |
| FES | Baseline | -.805 | 0.112 | -1.025 | | -0.584 | |
|  | Follow-up | -.572 | 0.113 | -0.794 | | -0.350 | |
| Attention |  |  |  |  | |  | |
| HC | Baseline | .045 | 0.110 | -0.172 | | 0.262 | |
|  | Follow-up | .297 | 0.110 | 0.080 | | 0.514 | |
| FES | Baseline | -.607 | 0.126 | -0.854 | | -0.360 | |
|  | Follow-up | -.869 | 0.128 | -1.122 | | -0.617 | |
| Psychomotor speed |  |  |  |  | |  | |
| HC | Baseline | .025 | 0.111 | -0.193 | | 0.243 | |
|  | Follow-up | .256 | 0.111 | 0.038 | | 0.474 | |
| FES | Baseline | -1.440 | 0.126 | -1.688 | | -1.191 | |
|  | Follow-up | -1.306 | 0.130 | -1.562 | | -1.050 | |
| Mental speed |  |  |  |  | |  | |
| HC | Baseline | -.013 | 0.126 | -0.262 | | 0.236 | |
|  | Follow-up | .021 | 0.127 | -0.229 | | 0.271 | |
| FES | Baseline | -1.351 | 0.142 | -1.631 | | -1.071 | |
|  | Follow-up | -1.398 | 0.147 | -1.688 | | -1.108 | |
| Working memory |  |  |  |  | |  | |
| HC | Baseline | .019 | 0.099 | -0.176 | | 0.214 | |
|  | Follow-up | .241 | 0.100 | 0.044 | | 0.438 | |
| FES | Baseline | -.923 | 0.112 | -1.143 | | -0.703 | |
|  | Follow-up | -.816 | 0.120 | -1.051 | | -0.580 | |
| Verbal fluency |  |  |  |  | |  | |
| HC | Baseline | -.035 | 0.090 | -0.212 | | 0.142 | |
|  | Follow-up | .356 | 0.091 | 0.177 | | 0.534 | |
| FES | Baseline | -1.365 | 0.103 | -1.567 | | -1.163 | |
|  | Follow-up | -.948 | 0.106 | -1.157 | | -0.739 | |
| Cognitive control |  |  |  |  | |  | |
| HC | Baseline | .017 | 0.134 | -0.247 | | 0.281 | |
|  | Follow-up | .420 | 0.135 | 0.155 | | 0.685 | |
| FES | Baseline | -.997 | 0.150 | -1.293 | | -0.701 | |
|  | Follow-up | -1.350 | 0.159 | -1.663 | | -1.037 | |
| Composite |  |  |  |  | |  | |
| HC | Baseline | .007 | 0.074 | -0.138 | | 0.153 | |
|  | Follow-up | .297 | 0.075 | 0.150 | | 0.444 | |
| FES | Baseline | -1.063 | 0.087 | -1.234 | | -0.892 | |
|  | Follow-up | -1.005 | 0.088 | -1.179 | | -0.831 | |

*Estimated marginal means (EM) from the mixed models with group comparisons of the effect of time for domain scores and composite scores, with standard error (SE) and a 95% confidence interval (CI). Age was included as a covariate in the models and evaluated at 29 years. HC: healthy controls; FES: participants with first-episode schizophrenia.*
